# Understanding *Phlebotomus perniciosus* abundance in south-east Spain: assessing the role of environmental and anthropic factors

**DOI:** 10.1186/s13071-017-2135-3

**Published:** 2017-04-19

**Authors:** José Risueño, Clara Muñoz, Pedro Pérez-Cutillas, Elena Goyena, Moisés Gonzálvez, María Ortuño, Luis Jesús Bernal, Juana Ortiz, Bulent Alten, Eduardo Berriatua

**Affiliations:** 1 Departamento de Sanidad Animal, Facultad de Veterinaria, Regional Campus of International Excellence “Campus Mare Nostrum”, Universidad de Murcia, 30100 Murcia, Spain; 2 Centro de Edafología y Biología Aplicada del Segura, Consejo Superior de Investigaciones Científicas (CEBAS-CSIC), 30100 Murcia, Spain; 3 Departamento de Geografía, Universidad de Murcia, 30001 Murcia, Spain; 4 Departamento de Medicina y Cirugía Animal, Facultad de Veterinaria, Regional Campus of International Excellence “Campus Mare Nostrum”, Universidad de Murcia, 30100 Murcia, Spain; 5 Department of Biology, Ecology section, Faculty of Science, Hacettepe University HU-ESRL-VERG laboratories, Ankara, Turkey

**Keywords:** *Phlebotomus perniciosus*, Abundance, Distribution, Environment, Climate, Leishmaniosis, Murcia, Spain

## Abstract

**Background:**

Leishmaniosis is associated with *Phlebotomus* sand fly vector density, but our knowledge of the environmental framework that regulates highly overdispersed vector abundance distributions is limited. We used a standardized sampling procedure in the bioclimatically diverse Murcia Region in Spain and multilevel regression models for count data to estimate *P. perniciosus* abundance in relation to environmental and anthropic factors.

**Methods:**

Twenty-five dog and sheep premises were sampled for sand flies using adhesive and light-attraction traps, from late May to early October 2015. Temperature, relative humidity and other animal- and premise-related data recorded on site and other environmental data were extracted from digital databases using a geographical information system. The relationship between sand fly abundance and explanatory variables was analysed using binomial regression models.

**Results:**

The total number of sand flies captured, mostly with light-attraction traps, was 3,644 specimens, including 80% *P. perniciosus*, the main *L. infantum* vector in Spain. Abundance varied between and within zones and was positively associated with increasing altitude from 0 to 900 m above sea level, except from 500 to 700 m where it was low. Populations peaked in July and especially during a 3-day heat wave when relative humidity and wind speed plummeted. Regression models indicated that climate and not land use or soil characteristics have the greatest impact on this species density on a large geographical scale. In contrast, micro-environmental factors such as animal building characteristics and husbandry practices affect sand fly population size on a smaller scale.

**Conclusions:**

A standardised sampling procedure and statistical analysis for highly overdispersed distributions allow reliable estimation of *P. perniciosus* abundance and identification of environmental drivers. While climatic variables have the greatest impact at macro-environmental scale, anthropic factors may be determinant at a micro-geographical scale. These finding may be used to elaborate predictive distribution maps useful for vector and pathogen control programs.

**Electronic supplementary material:**

The online version of this article (doi:10.1186/s13071-017-2135-3) contains supplementary material, which is available to authorized users.

## Background

Phlebotomine sand flies (Diptera: Psychodidae) are haematophagous insects that transmit *Leishmania* spp., protozoan parasites endemic in tropical and temperate zones, including the Mediterranean subregion [1]. Among over 800 sand fly species worldwide, 12 have been identified in Spain [2]. These include *Phlebotomus perniciosus* and *P. ariasi*, vectors of *Leishmania infantum* responsible for zoonotic visceral leishmaniosis in western Mediterranean countries; and *P. papatasi* and *P. sergenti*, vectors of *L. major* and *L. tropica*, respectively, that cause cutaneous leishmaniosis in Northern Africa and the Middle East.

The risk of *L. infantum* infection in endemic areas is geographically variable, depending on sand fly density [3, 4]. Unlike mosquitoes, sand flies breed on terrestrial sites protected from desiccation and with organic matter for larvae to feed on such as animal burrows and shelters, abandoned buildings, caves and stone walls [5]. Efforts to collect immature sand fly stages from the natural environment are very unproductive, and the precise microhabitats for sand fly breeding are poorly characterised [6]. Hence, the great majority of sand fly distribution studies monitor adult stages only. Their activity is typically seasonal; they can be found over a broad altitudinal range and temperature is considered the main artifice of sand fly phenology patterns in Mediterranean countries [4]. The role of other climatic and environmental variables on sand fly abundance is still inconclusive due to the wide variety of natural habitats in which sand flies are found, and the complex interconnections between the multiple factors affecting sand fly biological cycles. Species may have preferential macrohabitats, and in western Europe *P. perniciosus* is widespread while *P. ariasi* prevails in cooler, more humid regions [2, 7, 8]. Locally, sand fly presence and abundance may vary depending on climate, orientation, predominant vegetation, soil types, proximity to livestock and other factors [6, 9–14]. Accurate mapping of sand fly densities is further constrained by the wide variability in study designs, sand fly collection methods and statistical methods used to analyse distributions. They are commonly collected using light-attraction and/or adhesive interception traps, and they may lead to significantly different sand fly density estimations [15, 16]. Complex data statistical analysis is required for quantitative longitudinal study designs that recognise the strong spatial and temporal aggregation, so typical of sand fly populations [4].

The Murcia Region in southeastern Spain is endemic for canine and human leishmaniosis caused by *L. infantum*. Recent studies have shown that asymptomatic infection is widespread in rural areas and prevalence is associated with specific environmental factors [17, 18]. There are no investigations of the spatial distribution of sand flies in most of the areas covered by the previous leishmaniosis studies. Surveys performed in areas close to Murcia City in the 1980s identified eight sand fly species and *P. perniciosus* was the most frequent vector, active between March and October, with peaks in July and September [19]. The Murcia Region is geographically and bioclimatically diverse due to its relatively large size (11,300 km^2^), altitudinal gradient (0–2,000 m above sea level) and distance range to the Mediterranean Sea (0–200 km). This makes it an ideal place for a quantitative investigation of environmental factors driving sand fly abundance on a large geographical scale. With this objective in mind, the present longitudinal study used a standardised sampling procedure to estimate sand fly abundance in rural areas in the main five bioclimatic zones in Murcia. Mixed generalised linear models for count data were then used to analyse overdispersed sand fly distributions in relation to macro/micro environmental and human-driven factors.

## Methods

### Study area and design

**Fig. 1:**
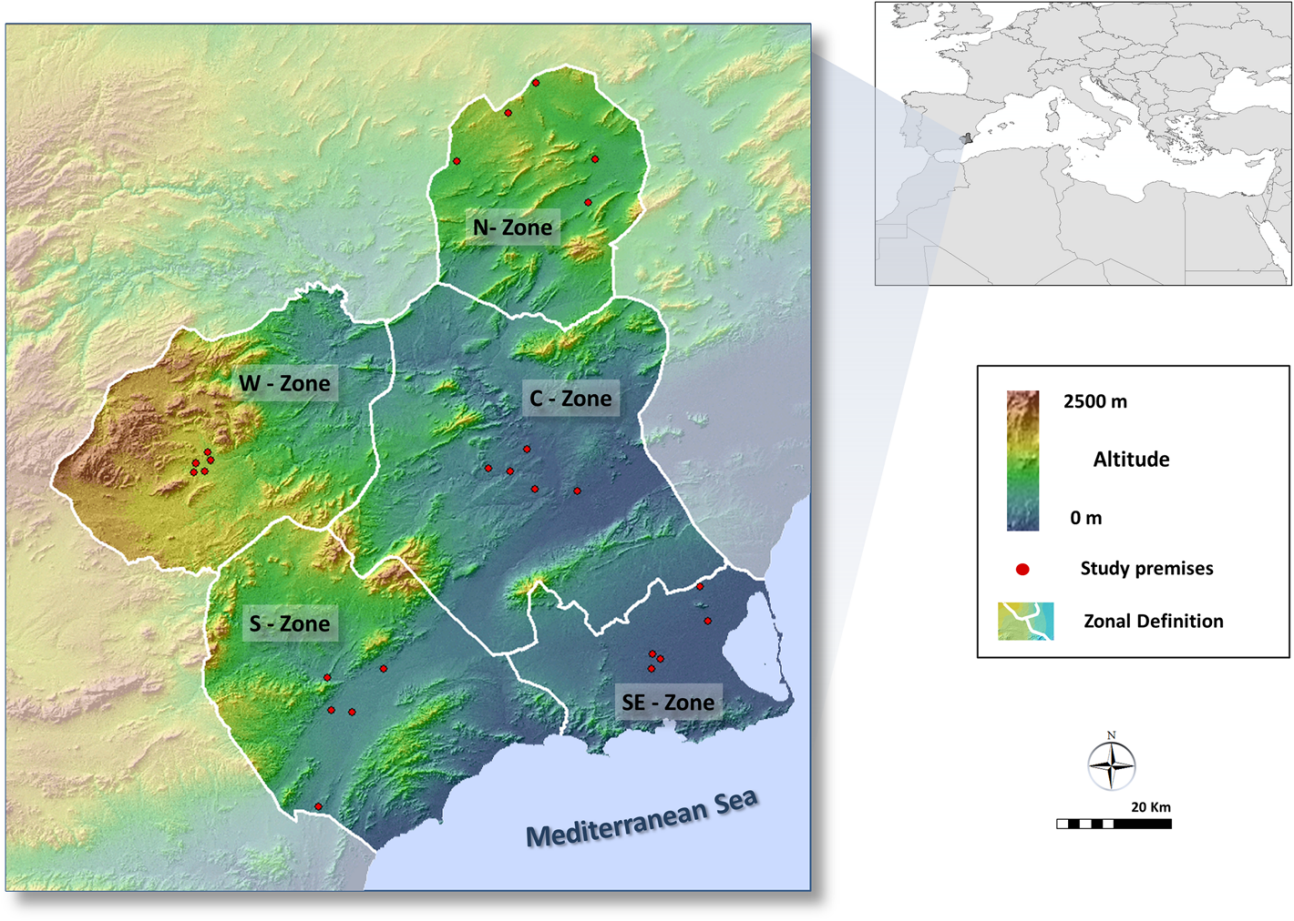
Location of study premises in Murcia Region, south-east Spain

The Murcia Region, with a permanent population of 1,470,000, has an agricultural and tourism based economy. It has a typical semi-arid Mediterranean climate, with long, dry summers and an average annual rainfall of 350 mm, which is commonly delivered over a few intense precipitation events. Sand fly abundance was monitored in 25 animal premises including 5 premises, 3 sheep sheds and 2 dog kennels, in each of the main five geographical zones that are traditionally recognized in the Murcia Region: N (north), S (south), C (central), W (west) and SE (southeast) (Fig. 1, Table 1). Premises were selected by local veterinarians based on owners’ willingness to participate and the prevalence of *Leishmania* infection in the animals was unknown. Premises in each zone were situated within a narrow altitude range and at a similar distance to the sea (Fig. 1, Table 1).

**Table 1:** Percentage of CDC traps with sandflies (positive traps) and sandfly abundance in positive traps

Zone	Premise	Altitude^a^ (m)	No. of traps	% positive traps	Abundance							
					Total	Mean	Min	25%	Median	75%	Max	*P*-value
Central (C)	Dog kennel 1	87	8	75	22	4	1	1	3	5	9	0.1081
	Dog kennel 2	207	8	75	33	6	3	4	5	7	9	
	Sheep flock 1	115	7	86	46	8	1	7	9	11	11	
	Sheep flock 2	145	8	50	14	4	2	2	2	4	8	
	Sheep flock 3	125	8	75	16	3	1	1	2	4	6	
	All		39	72	131	5	1	2	4	8	11	
North (N)	Dog kennel 1	629	8	50	14	4	2	3	3	4	6	0.0083
	Dog kennel 2	536	8	25	2	1	1	1	1	1	1	
	Sheep flock 1	660	8	75	156	26	10	13	17	23	76	
	Sheep flock 2	705	8	75	40	7	2	4	5	5	20	
	Sheep flock 3	794	8	25	5	3	2	2	3	3	3	
	All		40	50	211	11	1	3	5	13	76	
South (S)	Dog kennel 1	352	8	100	399	50	10	19	56	70	92	0.0042
	Dog kennel 2	265	8	38	7	2	1	2	2	3	4	
	Sheep flock 1	291	7	100	561	80	4	21	44	123	225	
	Sheep flock 2	322	7	100	132	19	2	7	11	32	41	
	Sheep flock 3	286	8	75	60	10	8	9	10	10	13	
	All		38	82	1159	37	1	10	13	46	225	
South-East (SE)	Dog kennel 1	55	7	43	5	2	1	2	2	2	2	0.1390
	Dog kennel 2	83	8	38	3	1	1	1	1	1	1	
	Sheep flock 1	25	7	86	17	3	1	2	3	4	5	
	Sheep flock 2	53	8	25	3	2	1	1	2	2	2	
	Sheep flock 3	44	8	13	1	1	1	1	1	1	1	
	All		38	39	29	2	1	1	2	2	5	
West (W)	Dog kennel 1	882	8	75	303	51	13	26	36	42	151	0.2701
	Dog kennel 2	889	7	86	189	32	1	11	18	50	84	
	Sheep flock 1	875	8	88	619	88	11	33	55	128	232	
	Sheep flock 2	882	7	71	247	49	3	13	19	51	161	
	Sheep flock 3	844	7	86	215	36	3	9	10	27	151	
	All		37	81	1,573	54	1	12	28	58	232	
All			192	65	3,109	25	1	3	9	22	232	<0.0001

^a^Metres above sea level

Every premise was sampled eight times for 24 h/time, at 2 week intervals between late May and mid-October 2015, except in August when it was not possible to collect samples. Sampling all 25 premises once took 1 week, from Monday to Friday. Sampling started on Monday 25th of May (4th week of May) and ended Friday 16th of October (3rd week of October).

Sand fly trapping devices included a miniature CDC (Centers for Disease Control and prevention) light attraction trap (J. W. Hock Company) and eight A4 castor oil impregnated white paper sheets (interception adhesive sticky traps) in each premise. The total number of light trap-days placed in the study were 200 (1 trap × 25 premises × 8 days) and similarly, the number of sticky trap-days was 1,600 (8 traps × 25 premises x 8 days). Traps were always placed in the same spot on each visit inside the building except for sticky traps in dog kennels where four were placed inside the dog house and the other half on in the open-air part outside the dog house. In all premises, sites selected for sticky traps were considered representative of different premise microhabitats, and they included wall surfaces and holes, fences and open windows. After each 24 h sampling, sticky traps and collection cups from light traps were gathered and taken back to the laboratory within a few hours.

### Sand fly counting, sexing and morphological speciation

Collection cups were kept at -20 °C for at least 2 h and sand flies were then counted, sexed and stored in absolute ethanol until speciated. Sticky traps were kept in the fridge and submitted to same procedure regularly and were completed by December 2015. Morphological identification was performed using entomological keys [20–22]. Briefly, the males were identified according to morphological features in the aedagus, stylopodite and coxopodite and the females were identified based on the pharynx, cibarium and spermatheca.

### Environmental data collection

A thermohygrometer (Digital Logtag Haxo-8 T, Templyzer) to record temperature and relative humidity (RH) was placed in each premise, inside the building, 2–3 m from the light traps, and measurements were taken every 3 h. Geographical coordinates were recorded using a global positioning system (GPS) devise. ArcGIS v.10 (ESRI, Redlands, USA) was used to map premises and delineate a 500 m buffer zone around them to extract environmental data for statistical analysis. Data included climatic information from the time series 2006–2015 from 56 weather stations was obtained through interpolation [23]. They included data from 47 stations in the Murcia Region http://siam.imida.es/) and 9 stations from neighboring regions (Castilla La Mancha: http://crea.uclm.es/siar/datmeteo/; Comunidad Valenciana: http://riegos.ivia.es/red-siar; Andalucía: http://www.juntadeandalucia.es/agriculturaypesca/ifapa/ria). Topographic and geomorphological data was derived from the digital elevation model (DEM) from TERRA mission, that uses the sensor Advanced Space-borne Thermal Emission and Reflection Radiometer (https://asterweb.jpl.nasa.gov/gdem.asp) and soil taxonomy from the LUCDEME project (http://www.magrama.gob.es/) (scale 1:100000). Ground classification data was obtained from Magna (http://info.igme.es/cartografia/magna50.asp) (scale 1:100000) and land use coverage, from CORINE Land Cover (scale 1:100000) (http://www.eea.europa.eu/data-and-maps/data/clc-2006-vector-data-version).

Climatic data from meteorological stations included daily averages for the study period and monthly averages for the 2006–2015 time series of the following variables: absolute maximum, maximum, mean, minimum and absolute minimum RH and temperature, maximum and total rainfall, and maximum and mean wind speed. Furthermore, annual, May-October (adult sand fly activity period in Murcia), November-April (period of null or low adults sand fly activity) mean values were calculated for climatic variables and used as independent variables in some of the multivariable regression models described below.

Data relating to the premises, animal management, structural features of the buildings, the frequency of use of disinfectants in the building and insecticides on the animals and position of the traps, were collected by inspecting and taking measurements of the building and interviewing the owner.

### Statistical analysis

The distribution of sand flies and environmental variables and the association between the presence/absence and sand fly counts in positive traps and other variables were analysed. Yates-corrected chi-squared test and the non-parametric Kruskal-Wallis test were used to compare proportions and medians, respectively, and the correlation between numerical variables was assessed using Spearman’s rank coefficient test. Multilevel negative binomial regression models were then developed to examine the independent contribution of environmental factors to sand fly abundance considering the correlation between repeated sand fly counts over time in study premises [24].

Two types of multilevel models were developed according to the data used: (i) Type I model used temperature and RH data from thermohygrometers, building characteristics and environmental data (other than temperature, RH and precipitation) from the buffer area around the premises, and (ii) Type II model used GIS-derived environmental data from the buffer area around the premises only. The later were developed to identify variables that could be used to generate a sand fly density map for Murcia Region and to compare outputs with *Leishmania* prevalence models. Environmental variables were used as fixed explanatory variables. They were fitted as categorical variables in the Type I models and as continuous variables in the Type II models. Random variation in sand fly counts between premises was considered both at the intercept and in the slope over time [24]. Briefly, this allows for variation between premises in the relationship between explanatory variables and the response (intercept variation), and for this variation to be different for each premise over time (slope variation).

A step-wise model building approach [25] was used beginning with a model including climatic variables. Other environmental variables significantly associated and showing a positive or negative trend with the outcome in the bivariate analysis, were subsequently added to the model. They included building characteristics, land use and soil and ground taxonomy variables. Due to the high correlation between environmental variables (for example between building age and type of wall material or altitude and temperature), several models including only variables significantly associated with sand fly counts were considered. Among them, the one with the lowest Akaike information criteria (AIC) were deemed the most parsimonious [25]. Parameter estimates were exponentiated to calculate incidence rate ratios. Significance was taken for alpha = 5% (*P* <0.05). R (http://cran.r-project.org/) program was used for all the statistical analysis.

## Results

### Overall sand fly abundance and species distribution

The total number of samples captured was 3,644 sand flies including 3,109 (85%) and 535 (15%) with CDC and sticky traps, respectively. The percentage of CDC and sticky traps with at least one sand fly (positive traps) were 65% (124/192) and 46% (91/198), respectively. The median (range) number of sand flies was 9 (1–232) in CDC traps (Table 1) and 6 (2–83) sand flies/m^2^ in sticky traps (Additional file 1: Table S1).

CDC and sticky traps provided a similar distribution of sand flies (Table 1 and Additional file 1: Table S1). The percentage of positive CDC traps and sand fly abundance in CDC traps were highest in the W and S zones and lowest in the SE (Table 1). Abundance in sticky traps was also greatest in the W and lowest in the SE, while the percentage of positive sticky traps was highest in the S and lowest in the SE (Additional file 1: Table S1). In addition to differences between zones, sand fly abundance also varied significantly between premises in the same zone, particularly in CDC traps from the S and the N (Table 1). Sand fly abundance was positively associated with altitude except that it was lower in the 536–705 m compared to 282–352 m altitude ranges (*P* < 0.05). These two ranges corresponded mostly, to premises located in the N and S of the region, respectively (Table 1)

The species distribution in the 3,586 (98%) sand flies speciated is shown in Table 2. *P. perniciosus* represented 80% of all sand flies followed by *P. papatasi* (10%), *P. sergenti* (5%), *S. minuta* (4%) and *P. chabaudi*, *P. longicuspis*, *P. ariasi* and *P. alexandri* (less than 1%) (Table 2). Species distribution varied according to trap type, zone and animal species premises. *Phlebotomus perniciosus* was relatively more abundant in CDC compared to sticky traps, in sheep than in dog premises and less abundant in the C zone compared to other zones (Table 2). Instead, the relative abundance of *P. papatasi* was greatest in sticky traps and C and N zones, *P. sergenti* in sticky traps, dog premises and W zone, and *S. minuta* in dog premises and in C (Table 2). The overall sex ratio was similar for all species (Table 2). However, in CDC traps *P. papatasi*, *P. sergenti* and *P. longicuspis* females were substantially more abundant than males while in sticky traps, 76% of all sand flies were males (not shown).

**Table 2:** Sandfly species absolute (relative) abundance according to explanatory variables

Variable	Level	Sand fly species								
		*P. perniciosus*	*P. papatasi*	*P. sergenti*	*S. minuta*	*P. chabaudi*	*P. longicuspis*	*P. ariasi*	*P. alexandri*	All
Trap	CDC	2,563 (84)	219 (7)	108 (4)	121 (4)	17 (1)	17 (1)	14 (1)	1 (< 1)	3,060 (100)
	Sticky	290 (55)	141 (27)	61 (12)	24 (5)	6 (1)	3 (1)	1 (< 1)	0 (0)	526 (100)
Zone	W	1,439 (82)	84 (5)	136 (8)	61 (3)	22 (1)	9 (1)	7 (< 1)	1 (< 1)	1,759 (100)
	SE	27 (87)	2 (2)	0 (0)	1 (3)	0 (0)	0 (0)	1 (3)	0 (0)	31 (100)
	S	1,041 (80)	146 (11)	29 (2)	62 (5)	1 (< 1)	8 (1)	7 (1)	0 (0)	1,296 (100)
	C	102 (55)	66 (36)	0 (0)	14 (8)	0 (0)	2 (1)	0 (0)	0 (0)	184 (100)
	N	244 (77)	62 (19)	4 (1)	7 (2)	0 (0)	1 (< 1)	0 (0)	0 (0)	318 (100)
Sex	Female	1,310 (79)	191 (12)	70 (4)	62 (4)	0 (0)	16 (1)	8 (1)	1 (< 1)	1,658 (100)
	Male	1,543 (80)	169 (9)	99 (5)	83 (4)	23 (1)	4 (< 1)	7 (< 1)	0 (0)	1,930 (100)
Premises	Dog kennel	824 (71)	91 (8)	108 (9)	103 (9)	20 (2)	8 (1)	13 (1)	1 (< 1)	1,168 (100)
	Sheep shed	2,029 (84)	269 (11)	61 (3)	42 (2)	3 (< 1)	12 (1)	2 (< 1)	0 (0)	2,420 (100)
Week	1	152 (78)	25 (13)	6 (3)	4 (2)	0 (0)	6 (3)	3 (2)	0 (0)	196 (100)
	2	491 (87)	58 (10)	6 (1)	7 (1)	1 (< 1)	1 (< 1)	2 (< 1)	0 (0)	566 (100)
	3	276 (82)	44 (13)	6 (2)	6 (2)	0 (0)	0 (0)	4 (1)	0 (0)	336 (100)
	4	789 (75)	111 (10)	75 (7)	56 (5)	15 (1)	9 (1)	3 (< 1)	1 (< 1)	1,059 (100)
	5	486 (73)	48 (7)	70 (10)	52 (8)	7 (1)	3 (< 1)	1 (< 1)	0 (0)	667 (100)
	6	479 (87)	59 (11)	4 (1)	8 (1)	0 (0)	1 (< 1)	2 (< 1)	0 (0)	553 (100)
	7	82 (78)	13 (12)	2 (2)	8 (8)	0 (0)	0 (0)	0 (0)	0 (0)	105 (100)
	8	98 (94)	2 (2)	0 (0)	4 (4)	0 (0)	0 (0)	0 (0)	0 (0)	104 (100)
All		2,853 (80)	360 (10)	169 (5)	145 (4)	23 (< 1)	20 (< 1)	15 (< 1)	1 (< 1)	3,586 (100)

### Seasonal distribution of *P. perniciosus* in CDC traps and bivariate relationship with indoor temperature and relative humidity

**Fig. 2:**
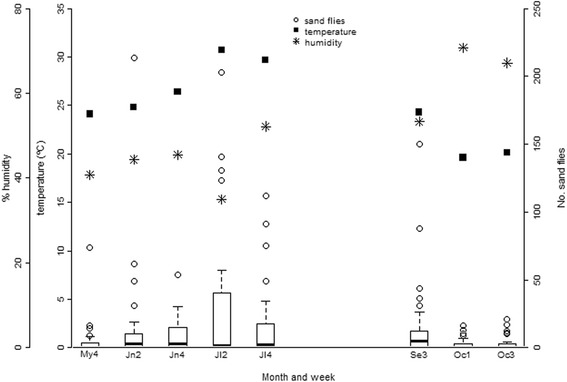
Seasonal CDC trap sand fly distribution and mean indoor temperature and humidity when traps were placed

**Fig. 3:**
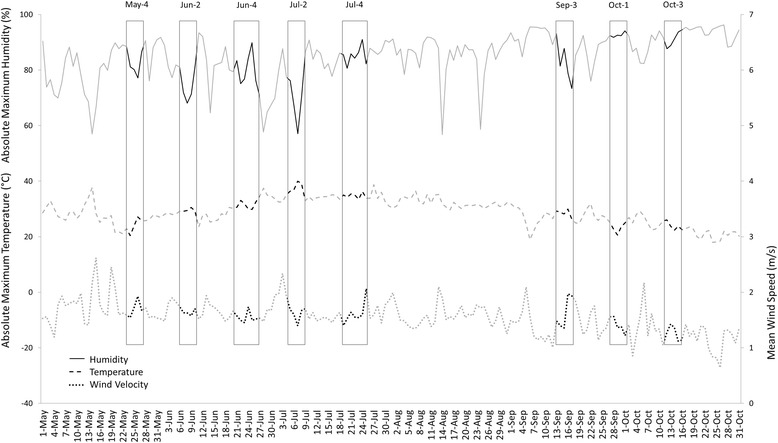
Mean absolute maximum temperature and humidity and wind speed in meteorological stations closest to premises

The spatio-temporal distribution of male and female *P. perniciosus* was similar (*P* > 0.05), and Fig. 2 shows the seasonal abundance for both sexes in CDC traps, together with the mean indoor-recorded temperature and RH, on the day when traps were collected. Abundance for this and other major species was highest in July and peaked sharply in all zones, during the second week of this month, and few sand flies were collected in May (week 1) and October (weeks 7 and 8) (Fig. 2, Table 2). The peak in *P. perniciosus* abundance in the week 2 of July coincided with the lowest recorded mean RH and highest mean temperature in the study (Fig. 2). This was associated with a similarly drastic change in the weather regionally, particularly the days when W, S and N were sampled; the mean maximum RH and temperature and mean wind speed in these zones were 89%, 33 °C and 2.1 m/s on July 3rd and 53%, 40 °C and 1.4 m/s on July 7th (Fig. 3).

The strong, negative association between *P. perniciosus* abundance and RH is further reflected in Table 3 showing the relationship between these two variables in the overall study data set. Moreover, altitude was negatively correlated with temperature (*r* = -0.21) and RH (*r* = -0.37) (*P* < 0.05).

**Table 3:** Percentage of *P. perniciosus* positive traps and its abundance, according to building humidity and temperature

Variable	Level	No. of traps	% positive traps	95% CI	*P*-value	Sand fly distribution in positive traps						
						Mean	Min	25%	Median	75%	100%	*P*-value
Humidity (%)	19–40	19	84	11–100	0.0014	61	2	9	41	115	203	< 0.0001
	41–50	50	72	12–84		22	1	3	14	21	214	
	51–60	28	64	13–82		27	1	5	8	33	150	
	61–70	33	58	14–74		6	1	1	2	9	21	
	71–86	35	34	15–50		3	1	1	2	3	10	
Temperature (°C)	14–20	34	38	10–55	0.0066	8	1	2	5	13	21	0.6247
	21–22	19	58	11–80		14	1	3	5	13	74	
	23–24	31	58	12–75		20	1	5	10	20	150	
	25–26	22	91	13–103		16	1	2	6	21	62	
	27–28	23	65	14–85		35	1	5	10	31	214	
	29–30	16	56	15–81		28	1	1	3	34	112	
	31–33	20	75	16–94		50	1	2	11	76	232	

*Abbreviation*: *95% CI* 95% confidence interval

### Bivariate relationship between *P. perniciosus* in CDC traps, trap positioning, building characteristics and animal species and husbandry

The proportion of positive traps and sand fly abundance in positive traps was greatest in older, small and not frequently disinfected buildings, with unplastered stone or brick walls and traditional ceilings made of cane and plaster, wood or bricks (Table 4). Sand fly abundance was also numerically greater in poorly ventilated buildings with straw or soil bedding. Neither the proportion of positive traps or sand fly abundance in positive traps was associated with animal species (sheep or dogs), average number of animals or animal density in the building or the use of insecticidal treatments on the animals (Table 4). Although sand fly abundance was associated with trap distance to the wall, the relationship did not follow a density positive or negative trend (Table 4).

**Table 4:** Percentage *P. perniciosus* positive traps and its abundance according to trap, building and animal features

Variable	Level	No. of traps	% positive traps	95% CI	*P*-value	Sand fly distribution in positive traps						
						Mean	Min	25%	Median	75%	100%	*P*-value
Building age (years)	2–20	40	50	35–65	0.0052	3	1	1	2	4	8	0.0001
	30–50	100	56	46–66		22	1	3	8	20	214	
	>100	36	83	71–96		31	1	3	11	35	203	
Inner roof/ceiling structure	Cane and plaster	30	83	70–97	0.0179	26	1	3	7	11	203	0.0336
	Brick	22	73	54–91		44	1	4	12	52	214	
	Metal	55	53	40–66		1	1	2	3	5	26	
	Wood	22	59	39–80		24	1	3	13	21	131	
	Concrete	47	49	35–63		17	1	1	10	20	112	
Wall structure	Unplastered stone	29	83	69–97	0.0006	37	1	3	15	49	203	0.0655
	Unplastered brick	39	69	55–84		9	1	3	7	10	57	
	Plastered brick	100	54	44–64		20	1	2	5	18	214	
	Plastered stone	8	13	−10–35		1	1	1	1	1	1	
Floor bedding	Concrete	69	57	45–68	0.5164	11	1	1	5	12	112	0.0490
	Straw/earth	107	63	53–72		27	1	3	8	19	214	
Building volume (m^3^)	16–209	45	78	66–90	0.0040	28	1	4	11	24	203	0.0059
	238–477	61	46	33–58		19	1	1	2	4	214	
	525–10,000	70	61	50–73		16	1	3	8	15	141	
Building volume (m^3^)/open area (m^2^)	1–9	60	72	60–83	0.0788	14	1	3	5	10	141	0.0009
	10–29	77	53	42–64		17	1	2	3	10	214	
	52–125	39	56	41–72		42	1	12	21	47	203	
Annual building disinfections	0–2	76	74	64–84	0.0397	19	1	3	8	16	141	< 0.0001
	3–8	61	59	47–71		3	1	1	2	4	8	
	24–365	39	51	36–67		40	1	7	17	45	214	
Main animal species in the building	Dogs	78	55	44–66	0.2760	17	1	2	5	21	112	0.4400
	Sheep	114	64	55–73		25	1	3	7	17	214	
Average no. of animals in the building	4–18	47	60	46–74	0.7381	25	1	3	14	35	112	0.1675
	35–65	53	66	53–79		34	1	2	5	35	214	
	95–175	53	64	51–77		12	1	2	5	10	131	
	325–900	31	55	37–72		15	1	3	7	9	141	
Animal density	0–0.07	48	54	40–68	0.5733	12	1	2	5	12	112	0.1244
	0.08–0.18	59	61	49–73		27	1	3	11	21	214	
	0.24–2.16	69	64	52–75		21	1	2	5	12	203	
Animal density/open area	<1–4	85	56	46–67	0.6104	12	1	1	5	9	141	0.0330
	6–14	46	63	49–77		31	1	2	11	39	203	
	21–480	45	64	50–78		25	1	4	10	19	214	
Annual insecticidal treatments on animals	0	69	72	62–83	0.0225	30	1	3	9	21	214	0.4198
	1–3	61	51	38–63		21	1	2	13	24	131	
	5–17	38	71	57–85		15	1	2	5	11	88	
Trap distance to the floor (cm)	50_135	39	49	33–64	0.0901	19	1	1	7	17	141	0.1736
	150_180	67	70	59–81		19	1	2	5	15	203	
	190_225	78	62	51–72		40	1	4	21	40	214	
Trap distance to the wall (cm)	20	115	64	56–73	0.5411	16	1	2	6	13	88	0.0341
	30–60	30	53	35–71		26	1	3	10	22	214	
	100– −600	39	62	46–77		15	1	1	3	7	131	
Trap minimum distance to animals (cm)	30–50	81	69	59–79	0.1040	15	1	2	5	15	112	0.2287
	100–400	103	56	47–66		30	1	2	10	27	214	

*Abbreviation*: *95% CI* 95% confidence interval

### Bivariate relationship between *P. perniciosus* abundance in CDC traps and external temperature, relative humidity, rainfall and wind speed

A summary table of climatic variables recorded at weather stations, showing a negative or positive association with the proportion of *P. perniciosus* positive traps and/or its abundance in positive traps is presented as supplementary material (Additional file 1: Table S2). Both the proportion of positive traps and abundance in positive traps were consistently negatively associated to May-October mean RH and maximum wind speed (Additional file 1: Table S2). Similar consistently negative associations were observed between the proportion of positive traps and the maximum annual and maximum and mean November-April wind speed and maximum annual rainfall, and between abundance in positive traps and May-October absolute maximum temperature. Other climatic variables were negatively associated with either the proportion of *P. perniciosus* positive traps or abundance, but the relationship was less consistent (Additional file 1: Table S2). In contrast, maximum RH in November-April was positively associated with sand fly abundance (Additional file 1: Table S2).

### Bivariate relationship between *P. perniciosus* abundance in CDC traps and land use, soil and ground types

After excluding significant associations between the percentage of *P. perniciosus* positive traps/abundance and land uses and soil and ground types present in comparatively small amounts (for example land used as urban fabric and coniferous forests), and those in which neither a consistent positive or negative trend with sand fly abundance was observed, results may be summarized as follows. The percentage of positive traps and abundance was greater in areas with moderate or large amounts of non-irrigated arable land, sparse vegetation and sandy grounds compared to areas with little or no amounts of these land types (Additional file 1: Table S3). The proportion of positive traps was positively associated with fluvisols grounds and abundance was negatively associated with coluvial soils and positively associated with petrocalcic xerosols (Additional file 1: Table S3).

### Multivariable relationship between *P. perniciosus* sand fly abundance and environmental variables

*Phlebotomus perniciosus* count data adequately fitted a negative binomial distribution (*P* > 0.05). The most parsimonious Type I model included a variable combining indoor temperature and RH and external mean maximum wind speed between May and October, and building age (Table 5). Incidence rate ratios (IRR) were greatest for lowest RH and highest temperature and decreased with increasing RH, reaching their lowest value for RH > 60% and lowest temperature < 22 °C. Moreover, IRR increased with decreasing wind speed and increasing building age (Table 5). It was not possible to include random effects in this model as this led to model convergence failure.

**Table 5:** Incidence rate ratios from a negative binomial distribution model of *P. perniciosus* CDC trap counts

Variable	Level	Rate ratio	95% CI	*P*-value
% RH; T(°C)^a^	19–40; 27–33	1.00	–	–
	19–40; 22–26	0.52	0.20–1.30	0.160
	41–60; 22–26	0.44	0.18–1.07	0.071
	41–60; 27–33	0.35	0.13–0.95	0.038
	61–79; 16–21	0.10	0.04–0.24	< 0.0001
	61–79; 22–26	0.17	0.06–0.49	0.001
	61–79; 27–33	0.10	0.02–0.40	0.001
Maximum wind speed (m/s)^b^	9.87–10.34	1.00	–	–
	7.99–8.18	11.87	5.03–28.01	< 0.0001
	8.33–9.01	3.27	1.19–8.97	0.021
Building age (years)	0–10	1.00	–	–
	30–50	6.01	2.83–12.77	< 0.0001
	> 100	9.64	3.94–23.59	< 0.0001

*Abbreviation*: *95% CI* 95% confidence interval

^a^Combined indoor relative humidity (RH %) and temperature (T)

^b^Mean maximum May-October wind speed

Among Type II models, the one with the lowest AIC indicated that sand fly abundance was negatively associated with precipitation, maximum temperature and maximum wind speed in May to October. The model revealed wide variation between premises in the sand fly count baseline (intercept) although this variability remained constant during the study (slope) (Table 6).

**Table 6:** Estimates of a multilevel negative binomial model of *P. perniciosus* CDC trap counts

Variable	Estimate	Standard error	*P*-value
Fixed components			
Intercept	56.663	10.255	< 0.0001
Precipitation^a^	-0.201	0.093	0.030
Maximum temperature (°C)^a^	-1.380	0.304	0.000
Maximum wind speed (m/S)^a^	-1.654	0.313	< 0.0001
Random effects	Standard deviation		
Premises (intercept)	1.1921		
Week (slope)	0.0613		

^a^Average data (May to October)

## Discussion

In a recent study investigating the presence/absence of *P. perniciosus* in southern Spain, the probability of finding sand flies increased with altitude up to 769–1,153 m, reflecting the positive association between the sand fly presence and temperature in this altitude range [26]. Sand fly abundance in the present study was similarly lowest in coastal areas and highest in the 844–849 m altitude range (W zone). However, it was significantly lower at 536–794 m (N zone), indicating that altitude or temperature alone, are inadequate predictors of sand fly abundance. RH, which was strongly, negatively associated with sand fly abundance, was similarly low in the N, W and S (265–352 m) zones but sand fly counts were much greater in W and S than in the N. Models greatly improved when maximum wind speed was fitted because wind exposure was highest and most variable in the N and SE (44–83 m) zones. Sand flies are poor fliers [15], and the wind may prevent them from entering buildings and probably generates drafts inside animal buildings, discouraging adult sand fly activity there.

Climate was responsible for the observed seasonality and the marked fluctuations in sand fly abundance over a short period. The huge increase in the second week of July coincided with a “heat wave” characterised by a sharp increase in temperature and a drop-in RH and wind speed. As far as we are aware, there are no previous reports of similar increases in sand fly abundance following heat waves typical of Mediterranean summers. Notwithstanding, Branco et al. [11] reported highest sand fly density in central Portugal associated to highest average monthly temperature, lowest RH and absence of strong wind. A similar relationship between temperature and RH and the abundance of the sand fly *Lutzomyia shannoni* was reported in Florida in the USA [27]. Rainfall and RH were also negatively associated with sand fly activity in other Mediterranean regions [10, 28, 29]. Although high RH is required by sand fly instars to develop and adult sand flies are very sensitive to desiccation [30], low RH favours adult activity, possibly during short spells in search of food.

Animal building age was also strongly associated with sand fly abundance. It indirectly accounted for several factors that impact on sand fly survival. Sand flies are very sensitive to disinfectants and insecticides, but they were not frequently used except in the most modern dog premises. Besides, old buildings with stone walls and accumulated organic matter are considered ideal for sand flies to breed and rest. They were also poorly ventilated, and carbon dioxide (CO_2_) is a strong attractant for blood-searching females [16].

The role of land use, soil and ground types on sand fly abundance remains unclear. Many such variables were associated with sand fly abundance in the univariate analysis, but in most cases, there was no evidence of a consistently positive or negative trend. Exceptions were increasing sparsely vegetated and non-irrigated arable land and petrocalcic xerosol ground and decreasing coluvial soils associated with greater sand fly counts. However, none of these variables was retained in the final multivariable model. This may not be surprising given the strong correlation between environmental variables. The wide variety of environments in which *P. perniciosus* can thrive suggests that its density on a large geographical scale depends more on climatic conditions than on specific terrains and land uses. This conclusion, however, may not be extended to other regions and species [31]. Moreover, multilevel models revealed considerable unexplained variation between study premises in the same zone, so clearly, microhabitat factors not accounted for in this study can have a profound effect on sand fly density.

The strong correlation between outdoor and indoor climatic variables allowed using the former to model sand fly abundance and may be used to generate and validate sand fly abundance density maps, and identify areas that require further studies of vector and pathogen distribution. In previous epidemiological studies on canine and human leishmaniosis in Murcia Region, seroprevalence in dogs was highest in the S, lowest in the N and variable in the SE [18]. Similarly, human PCR prevalence was highest in the S zone, lowest in the N zone and C and variable in the SE zone [17]. Murcia coastal SE is climatically variable, and this could be associated with a higher sand fly and leishmaniosis spatial overdispersion. Leishmaniosis *foci* associated to *P. perniciosus* have been reported in coastal areas in Italy [32]. Further entomological and epidemiological studies are needed to in Murcia’s SE zone, as well as in the C and W zones where information on *Leishmania* prevalence is presently incomplete.

The study focused mainly on CDC light trap captures after observing that sand fly distributions in sticky traps were similar but had comparatively few sand flies. *P. perniciosus* was the most abundant species in both trap types. Light traps are particularly suited for sand flies with strong phototropism such as *P. perniciosus* females [32]. In contrast, sticky traps sample sand flies by interception providing an unbiased estimate of insect activity in a place [15]. *Sergentomyia minuta* feeds on reptiles and are not strongly phototropic and was the dominant species in most studies in Spain using sticky traps (reviewed by Galvez et al. [10]). However, the number species identified in the present study was the same and their relative abundance in light traps similar, to that reported in south-east Spain 30 years ago [33–36]. According to the later author, less common sand fly species have narrower preferential bioclimatic conditions and among them, *P. papatasi*, *P. sergenti* and *P. alexandri* favour arid zones [35]. While no definite conclusions can be drawn from the present study in this respect, *P. papatasi* was relatively more abundant in the most arid C zone, but *P. sergenti* was most common in the least arid W zone.

In summary, this study confirms the presence of sand flies in the Murcia Region including the two main *L. infantum* vectors, *P. perniciosus* and *P. ariasi*, and provides a quantitative analysis of their spatial distribution in relation to environmental variables. Sand fly abundance is heterogeneously distributed, strongly depending on temperature, RH, rainfall, wind speed and microenvironmental factors. These findings may be extrapolated to other Mediterranean regions to improve our understanding of *P. perniciosus* and *L. infantum* infection dynamics. Moreover, the methods used in this study may be a model to perform standardised and optimized abundance studies on sand flies.

## Conclusions

*Phlebotomus perniciosus* is the predominant sand fly species in the countryside in the Murcia Region, and its abundance is spatially and temporally heterogeneous. Climate, including relative humidity, temperature and wind speed and not land use or soil characteristics, have the greatest impact on sand fly density on a large geographical scale. Microenvironmental factors such as animal building characteristics and husbandry practices can significantly affect sand fly counts on a small geographical scale. These finding may be used to developing predictive vector and pathogen distribution maps.

## Additional file

Additional file 1: Table S1. Percentage of “sticky” traps with sandflies (positive traps) and sand fly abundance in positive traps in dog kennels and sheep flocks in Murcia Region, southeast Spain in 2015. **Table S2.** Percentage of CDC traps with *P. perniciosus* and abundance in positive traps according to climatic variables from meteorological stations. A study of sand fly abundance in dog kennels and sheep flocks in Murcia Region, southeast Spain, in 2015. **Table S3**. Percentage of CDC traps with *P. perniciosus* and abundance in positive traps according to land use and soil and ground types. A study of sand fly abundance in dog kennels and sheep flocks in Murcia Region, southeast Spain, in 2015. (DOC 209 kb)

## Abbreviations

AIC: Akaike information criteria
CDC: Centers for Disease Control and Prevention
DEM: Digital elevation model
GPS: Global positioning system
IRR: Incidence rate ratios
RH: Relative humidity

## References

[1] Lawyer PG, Perkins PV. Leishmaniasis and trypanosomiasis. In: Eldridge BF, Edman JD, editors. Medical entomology. Dordrecht: Kluwer Academic Publishers; 2000.

[2] Aransay AM, Testa JM, Morillas-Marquez F, Lucientes J, Ready PD (2004). Distribution of sand fly species in relation to canine leishmaniasis from the Ebro Valley to Valencia, northeastern Spain. Parasitol Res.

[3] Arce A, Estirado A, Ordobas M, Sevilla S, García N, Moratilla L (2013). Re-emergence of leishmaniasis in Spain: community outbreak in Madrid, Spain, 2009 to 2012. Eurosurveillance.

[4] Alten B, Maia C, Afonso MO, Campino L, Jiménez M, González E (2016). Seasonal dynamics of phlebotomine sand fly species proven vectors of Mediterranean leishmaniasis caused by *Leishmania infantum*. PLoS Negl Trop Dis.

[5] Feliciangeli MD (2004). Natural breeding places of phlebotomine sandflies. Med Vet Entomol.

[6] Killick-Kendrick R (1999). The biology and control of phlebotomine sand flies. Clin Dermatol.

[7] Ballart C, Barón S, Alcover MM, Portús M, Gállego M (2012). Distribution of phlebotomine sandflies (Diptera: Psychodidae) in Andorra: first finding of *P. perniciosus* and wide distribution of *P. ariasi*. Acta Trop.

[8] Rioux JA, Carron S, Dereure J, Périères J, Zeraia L, Franquet E (2013). Ecology of leishmaniasis in the south of France. 22. Reliability and representativeness of 12 *Phlebotomus ariasi*, *P. perniciosus* and *Sergentomyia minuta* (Diptera: Psychodidae) sampling stations in Vallespir (eastern French Pyrenees region). Parasite.

[9] Rioux JA, Golvan YJ, Croset H, Houin R (1969). Leishmanioses in the Mediterranean “Midi”: results of an ecologic survey. Bull Soc Pathol Exot Filiales.

[10] Gálvez R, Descalzo MA, Miró G, Jiménez MI, Martín O, Dos Santos-Brandao F (2010). Seasonal trends and spatial relations between environmental/meteorological factors and leishmaniosis sand fly vector abundances in central Spain. Acta Trop.

[11] Branco S, Alves-Pires C, Maia C, Cortes S, Cristovão JMS, Gonçalves L, Campino L, Afonso MO (2013). Entomological and ecological studies in a new potential zoonotic leishmaniasis focus in Torres Novas municipality, Central Region, Portugal. Acta Trop.

[12] Maroli M, Feliciangeli MD, Bichaud L, Charrel RN, Gradoni L (2013). Phlebotomine sandflies and the spreading of leishmaniases and other diseases of public health concern. Med Vet Entomol.

[13] Barón SD, Morillas-Márquez F, Morales-Yuste M, Díaz-Sáez V, Gállego M, Molina R, Martín-Sánchez J (2013). Predicting the risk of an endemic focus of *Leishmania tropica* becoming established in south-western Europe through the presence of its main vector, *Phlebotomus sergenti* Parrot, 1917. Parasitology.

[14] Alcover MM, Ballart C, Martín-Sánchez J, Serra T, Castillejo S, Portús M, Gállego M (2014). Factors influencing the presence of sandflies in Majorca (Balearic Islands, Spain) with special reference to *Phlebotomus pernicious*, vector of *Leishmania infantum*. Parasit & Vectors.

[15] Alexander B (2000). Sampling methods for phlebotomine sandflies. Med Vet Entomol.

[16] Alten B, Ozbel Y, Ergunay K, Kasap OE, Cull B, Antoniou M (2015). Sampling strategies for phlebotomine sandflies (Diptera: Psychodidae) in Europe. Bull Entomol Res.

[17] Pérez-Cutillas P, Goyena E, Chitimia L, De la Rúa P, Bernal LJ, Fisa R (2015). Spatial distribution of human asymptomatic *Leishmania infantum* infection in southeast Spain: a study of environmental, demographic and social risk factors. Acta Trop.

[18] Goyena E, Pérez-Cutillas P, Chitimia L, Risueño J, García-Martínez JD, Bernal LJ, Berriatua E (2016). A cross-sectional study of the impact of regular use of insecticides in dogs on canine leishmaniosis seroprevalence in southeast Spain. Prev Vet Med.

[19] Martínez Ortega E, Conesa Gallego E (1987). Fenología de los flebotomos del subgénero *Larrossius* (Diptera, Psychodidae, *Phlebotomus*) en el sureste de la Península Ibérica. Bol Asoc Esp Entomol.

[20] Lewis DJ (1982). A taxonomic review of the genus *Phlebotomus* (Diptera: Psychodidae) Bull Br Mus. Nat Hist.

[21] Gállego Berenguer J, Botet J, Gállego M, Portús M. Los flebotomos de la España peninsular e Islas Baleares. Identificación y corología. Comentarios sobre los métodos de captura. In: Hernández S, editor. In memoriam al Prof. Doctor. D. F. de P. Martínez Gómez. Córdoba: Publicaciones de la Universidad de Córdoba; 1992. p. 581–600.

[22] Lawyer P, Rowton E, Westbrooke K (2011). Recognition, indentification, mounting and dissection of Phlebotomine sand flies.

[23] Ustrnul Z, Czekierda D (2005). Application of GIS for the development of climatological air temperature maps: an example from Poland. Meteorol Appl.

[24] Snijders TAB, Bosker RJ (1999). Multilevel analysis: an Introduction to basic and advanced multilevel modeling.

[25] Kleinbaum DG, Kupper LL, Muller KE, Nizam A (1998). Applied regression analysis and other multivariable methods.

[26] Barón SD, Morillas-Márquez F, Morales-Yuste M, Díaz-Sáez V, Irigaray C, Martín-Sánchez J (2011). Risk maps for the presence and absence of *Phlebotomus perniciosus* in an endemic area of leishmaniasis in southern Spain: implications for the control of the disease. Parasitology.

[27] Mann RS, Kaufman PE (2010). The seasonal abundance of phlebotomine sand flies, *Lutzomyia* species in Florida. J Am Mosq Control Assoc.

[28] Dantas-Torres F, Tarallo VD, Latrofa MS, Falchi A, Lia RP, Otranto D (2014). Ecology of phlebotomine sand flies and *Leishmania infantum* infection in a rural area of southern Italy. Acta Trop.

[29] Prudhomme J, Rahola N, Toty C, Cassan C, Roiz D, Vergnes B (2015). Ecology and spatiotemporal dynamics of sand flies in the Mediterranean Languedoc region (Roquedur area, Gard, France). Parasit Vectors.

[30] Volf P, Volfova V (2011). Establishment and maintenance of sand fly colonies. J Vector Ecol.

[31] Colacicco-Mayhugh MG, Masuoka PM, Grieco JP (2010). Ecological niche model of *Phlebotomus alexandri* and *P. papatasi* (Diptera: Psychodidae) in the Middle East. Int J Health Geogr.

[32] Rossi E, Rinaldi L, Musella V, Veneziano V, Carbone S, Gradoni L (2007). Mapping the main *Leishmania* phlebotomine vector in the endemic focus of the Mt. Vesuvius in southern Italy. Geospat Health.

[33] Martínez-Ortega E, Romera H, Conesa-Gallego E, Goyena M (1991). Estudio comparado de la antropofilia y el fototropismo de los flebotomos en un foco de leishmaniasis del sureste de la Península Ibérica. Parassitologia (Roma).

[34] Martínez-Ortega E (1985). Los flebotomos ibéricos (díptera *Psychodidae*). I. Almeria. An Biol.

[35] Martínez-Ortega E (1985). Los flebotomos ibéricos (díptera *Psychodidae*). II. Sureste. An Biol.

[36] Martínez-Ortega E (1986). Biología de los flebotomos ibéricos (Diptera: Psychodidae) en condiciones naturales. Ann Ist Super Sanita.

