# Supplementary material for: Understanding Phlebotomus perniciosus abundance in south-east Spain: assessing the role of environmental and anthropic factors
Source: Parasit Vectors. 2017 Apr 19;10:189. doi: 10.1186/s13071-017-2135-3 (PMC5395901; doi:10.1186/s13071-017-2135-3)
Supplement: Additional file 1: Table S1. — Percentage of “sticky” traps with sandflies (positive traps) and sand fly abundance in positive traps in dog kennels and sheep flocks in Murcia Region, southeast Spain in 2015. Table S2. Percentage of CDC traps with P. perniciosus and abundance in positive traps according to climatic variables from meteorological stations. A study of sand fly abundance in dog kennels and sheep flocks in Murcia Region, southeast Spain, in 2015. Table S3. Percentage of CDC traps with P. perniciosus and abundance in positive traps according to land use and soil and ground types. A study of sand fly abundance in dog kennels and sheep flocks in Murcia Region, southeast Spain, in 2015. (DOC 209 kb) [file 13071_2017_2135_MOESM1_ESM.doc]

**Additional file 1**

**Additional file 1: Table S1.** Percentage of “sticky” traps with sandflies (positive traps) and sandfly abundance in positive traps in dog kennels and sheep flocks in Murcia Region, southeast Spain in 2015.

| Zone | Premises | Altitude  a. s. l. (m)* | No.  traps | % positive  traps | Abundance | | | | | | | |
| --- | --- | --- | --- | --- | --- | --- | --- | --- | --- | --- | --- | --- |
|  |  |  |  |  | total | mean | min | 25% | median | 75% | max | P value |
| East | dog kennel 1 | 87 | 8 | 50 | 20 | 5 | 2 | 4 | 15 | 24 | 25 | 0.3710 |
|  | dog kennel 2 | 207 | 8 | 38 | 5 | 2 | 2 | 3 | 4 | 5 | 5 |  |
|  | sheep flock 1 | 115 | 8 | 75 | 10 | 2 | 2 | 2 | 3 | 5 | 9 |  |
|  | sheep flock 2 | 145 | 8 | 38 | 17 | 6 | 2 | 2 | 2 | 18 | 34 |  |
|  | sheep flock 3 | 125 | 8 | 38 | 3 | 1 | 2 | 2 | 2 | 2 | 2 |  |
|  | All |  | 40 | 48 | 55 | 3 | 2 | 2 | 2 | 5 | 34 |  |
| North | dog kennel 1 | 629 | 8 | 63 | 41 | 8 | 2 | 4 | 5 | 14 | 62 | 0.2438 |
|  | dog kennel 2 | 536 | 8 | 50 | 11 | 3 | 2 | 2 | 3 | 7 | 14 |  |
|  | sheep flock 1 | 660 | 8 | 50 | 22 | 6 | 8 | 10 | 11 | 13 | 14 |  |
|  | sheep flock 2 | 705 | 8 | 63 | 13 | 3 | 2 | 4 | 4 | 8 | 8 |  |
|  | sheep flock 3 | 794 | 8 | 50 | 17 | 4 | 6 | 8 | 8 | 9 | 12 |  |
|  | All |  | 40 | 55 | 104 | 5 | 2 | 4 | 8 | 12 | 62 |  |
| South (S) | dog kennel 1 | 352 | 8 | 100 | 23 | 3 | 2 | 6 | 7 | 8 | 10 | 0.4775 |
|  | dog kennel 2 | 265 | 8 | 13 | 3 | 3 | 6 | 6 | 6 | 6 | 6 |  |
|  | sheep flock 1 | 291 | 8 | 75 | 74 | 12 | 2 | 12 | 35 | 47 | 53 |  |
|  | sheep flock 2 | 322 | 8 | 88 | 52 | 7 | 2 | 3 | 5 | 25 | 46 |  |
|  | sheep flock 3 | 286 | 8 | 50 | 10 | 3 | 2 | 4 | 4 | 5 | 7 |  |
|  | All |  | 40 | 65 | 162 | 6 | 2 | 4 | 6 | 10 | 53 |  |
| S. East | dog kennel 1 | 55 | 7 | 14 | 1 | 0 | 2 | 2 | 2 | 2 | 2 | - |
|  | dog kennel 2 | 83 | 8 | 0 | 0 | 0 | 0 | 0 | 0 | 0 | 0 |  |
|  | sheep flock 1 | 25 | 8 | 38 | 3 | 1 | 2 | 2 | 2 | 2 | 2 |  |
|  | sheep flock 2 | 53 | 8 | 0 | 0 | 0 | 0 | 0 | 0 | 0 | 0 |  |
|  | sheep flock 3 | 44 | 8 | 0 | 0 | 0 | 0 | 0 | 0 | 0 | 0 |  |
|  | All |  | 39 | 10 | 4 | 1 | 2 | 2 | 2 | 2 | 2 |  |
| West | dog kennel 1 | 882 | 8 | 50 | 40 | 10 | 4 | 9 | 13 | 25 | 50 | 0.8249 |
|  | dog kennel 2 | 889 | 7 | 57 | 65 | 16 | 5 | 12 | 32 | 54 | 66 |  |
|  | sheep flock 1 | 875 | 8 | 75 | 63 | 11 | 3 | 8 | 14 | 25 | 83 |  |
|  | sheep flock 2 | 882 | 8 | 38 | 15 | 5 | 4 | 8 | 12 | 13 | 14 |  |
|  | sheep flock 3 | 844 | 8 | 38 | 27 | 9 | 7 | 10 | 13 | 26 | 38 |  |
|  | All |  | 39 | 51 | 210 |  | 3 | 7 | 14 | 31 | 83 |  |
| All |  |  | 198 | 46 | 535 | 6 | 2 | 3 | 6 | 14 | 83 | 0.0219 |

* Premises altitude above sea level

**Additional file 1: Table S2.** Percentage of CDC traps with *P. perniciosus* and abundance in positive traps according to climatic variables from meteorological stations. A study of sandfly abundance in dog kennels and sheep flocks in Murcia Region, southeast Spain, in 2015

| Variable | Level  (% range) | No.  traps | % positive  traps | 95% CI | | | P value | Sandfly distribution in positive traps | | | | | | |
| --- | --- | --- | --- | --- | --- | --- | --- | --- | --- | --- | --- | --- | --- | --- |
|  |  |  |  |  | |  |  | Mean | Min. | 25% | median | 75% | 100% | P value |
| Relative humidity (%) |  |  |  |  | |  |  |  |  |  |  |  |  |  |
| May-October mean | 57.8-59.0 | 60 | 75 | 64 | | 86 | 0.0019 | 53 | 1 | 10 | 17 | 50 | 203 | <0.0001 |
|  | 59.1-61.3 | 86 | 63 | 53 | | 73 |  | 22 | 1 | 3 | 7 | 18 | 214 |  |
|  | 65.5-74.3 | 38 | 39 | 24 | | 55 |  | 2 | 1 | 1 | 1 | 2 | 5 |  |
| November-April maximum | 84.4-87.0 | 31 | 52 | 34 | | 69 | 0.0567 | 3 | 1 | 1 | 2 | 5 | 10 | 0.0009 |
|  | 87.1-88.0 | 39 | 67 | 52 | | 81 |  | 17 | 1 | 3 | 6 | 18 | 88 |  |
|  | 88.1-90.0 | 62 | 53 | 41 | | 66 |  | 22 | 1 | 2 | 7 | 15 | 214 |  |
|  | 90.1-93.1 | 52 | 75 | 63 | | 87 |  | 35 | 1 | 3 | 13 | 38 | 203 |  |
| Annual minimum | 36.7-38.0 | 37 | 81 | 68 | | 94 | 0.0027 | 44 | 1 | 11 | 21 | 49 | 203 | <0.0001 |
|  | 38.1-41.0 | 71 | 62 | 51 | | 73 |  | 13 | 1 | 2 | 7 | 11 | 88 |  |
|  | 41.1-43.0 | 38 | 66 | 51 | | 81 |  | 25 | 1 | 3 | 5 | 19 | 214 |  |
|  | 43.1-51.2 | 38 | 39 | 24 | | 55 |  | 2 | 1 | 1 | 1 | 2 | 5 |  |
| Wind speed (m/s) |  |  |  | |  |  |  |  |  |  |  |  |  |  |
| May-October maximum | 8.0-8.3 | 82 | 76 | | 66 | 85 | 0.0001 | 32 | 1 | 4 | 12 | 31 | 214 | <0.0001 |
|  | 8.4-8.7 | 40 | 70 | | 56 | 84 |  | 19 | 2 | 5 | 10 | 17 | 88 |  |
|  | 8.8-9.2 | 24 | 38 | | 18 | 57 |  | 2 | 1 | 1 | 2 | 3 | 5 |  |
|  | 9.3-10.4 | 38 | 39 | | 24 | 55 |  | 2 | 1 | 1 | 1 | 2 | 5 |  |
| November-April maximum | 9.7-10.3 | 53 | 75 | | 65 | 86 | 0.0000 | 22 | 1 | 3 | 7 | 18 | 214 | <0.0001 |
|  | 10.4-11.0 | 61 | 75 | | 64 | 87 |  | 37 | 1 | 10 | 17 | 42 | 203 |  |
|  | 11.1-11.8 | 24 | 43 | | 29 | 58 |  | 3 | 1 | 1 | 2 | 4 | 14 |  |
|  | 11.9-12.4 | 46 | 33 | | 14 | 52 |  | 2 | 1 | 1 | 2 | 2 | 4 |  |
| Annual maximum | 8.9-9.3 | 53 | 75 | | 64 | 87 | 0.0005 | 24 | 1 | 3 | 7 | 23 | 214 | <0.0001 |
|  | 9.4-9.8 | 61 | 72 | | 61 | 83 |  | 34 | 1 | 9 | 16 | 37 | 203 |  |
|  | 9.9-10.5 | 24 | 46 | | 26 | 66 |  | 4 | 1 | 1 | 3 | 5 | 14 |  |
|  | 10.6-11.1 | 46 | 41 | | 27 | 56 |  | 2 | 1 | 1 | 2 | 2 | 5 |  |
| November-April mean | 0.8-1.6 | 54 | 76 | | 65 | 87 | 0.0048 | 24 | 1 | 3 | 8 | 22 | 214 | 0.2488 |
|  | 1.7-2.4 | 106 | 60 | | 51 | 70 |  | 24 | 1 | 2 | 9 | 21 | 203 |  |
|  | 2.5-3.3 | 24 | 38 | | 18 | 57 |  | 4 | 1 | 2 | 3 | 5 | 14 |  |
| Rain fall (mm) |  |  |  | |  |  |  |  |  |  |  |  |  |  |
| Annual maximum | 12.4-13.0 | 77 | 69 | | 58 | 79 | 0.0057 | 22 | 1 | 4 | 9 | 18 | 214 | <0.0001 |
|  | 13.1-14.0 | 69 | 67 | | 56 | 78 |  | 30 | 1 | 3 | 11 | 31 | 203 |  |
|  | 14.1-15.2 | 38 | 39 | | 24 | 55 |  | 2 | 1 | 1 | 1 | 2 | 5 |  |
| Temperature (ºC) |  |  |  | |  |  |  |  |  |  |  |  |  |  |
| May-October absolute | 32.4, 34.0 | 67 | 63 | | 51 | 74 | 0.7130 | 32 | 1 | 2 | 13 | 35 | 203 | 0.0007 |
| maximum | 34.1, 35.0 | 78 | 64 | | 53 | 75 |  | 23 | 1 | 3 | 9 | 19 | 214 |  |
|  | 35.1, 36.8 | 39 | 56 | | 41 | 72 |  | 4 | 1 | 1 | 3 | 5 | 10 |  |
| May-October maximum | 24.1, 25.0 | 37 | 81 | | 68 | 94 | 0.0046 | 44 | 1 | 11 | 21 | 49 | 203 | 0.0000 |
|  | 25.1, 26.0 | 78 | 50 | | 39 | 61 |  | 12 | 1 | 2 | 3 | 9 | 88 |  |
|  | 26.1, 27.3 | 69 | 65 | | 54 | 76 |  | 17 | 1 | 1 | 5 | 15 | 214 |  |
| Annual absolute maximum | 26.7, 28.0 | 37 | 81 | | 68 | 94 | 0.0022 | 44 | 1 | 11 | 21 | 49 | 203 | 0.0022 |
|  | 28.1, 29.0 | 77 | 49 | | 38 | 61 |  | 18 | 1 | 2 | 4 | 14 | 214 |  |
|  | 29.1, 30.0 | 31 | 77 | | 63 | 92 |  | 19 | 1 | 4 | 9 | 21 | 88 |  |
|  | 30.1, 31.6 | 39 | 56 | | 41 | 72 |  | 4 | 1 | 1 | 3 | 5 | 10 |  |
| Annual maximum | 18.8, 20.0 | 53 | 72 | | 60 | 84 | 0.08386 | 36 | 1 | 6 | 15 | 38 | 203 | <0.0001 |
|  | 20.1, 21.0 | 47 | 68 | | 55 | 81 |  | 18 | 1 | 5 | 10 | 18 | 88 |  |
|  | 21.1, 22.0 | 45 | 49 | | 34 | 63 |  | 24 | 1 | 1 | 2 | 12 | 214 |  |
|  | 22.1, 22.4 | 39 | 56 | | 41 | 72 |  | 4 | 1 | 1 | 3 | 5 | 10 |  |
| November-April absolute | -3.3, -2.0 | 48 | 70 | | 59 | 80 | 0.0101 | 31 | 1 | 5 | 14 | 32 | 203 | 0.0000 |
| minimum | -1.9, 1.0 | 22 | 71 | | 55 | 87 |  | 37 | 1 | 12 | 34 | 62 | 88 |  |
|  | 1.1, 2.0 | 25 | 66 | | 51 | 81 |  | 25 | 1 | 5 | 8 | 18 | 214 |  |
|  | 2.1, 2.7 | 19 | 41 | | 27 | 56 |  | 2 | 1 | 1 | 1 | 3 | 10 |  |

**Additional file 1: Table S3.** Percentage of CDC traps with *P. perniciosus* and abundance in positive traps according to land use and soil and ground types. A study of sandfly abundance in dog kennels and sheep flocks in Murcia Region, southeast Spain, in 2015

| Variable | Level  (% range) | No.  traps | % positive  traps | Confidence  interval | | P value | Sandfly distribution in positive traps | | | | | | |
| --- | --- | --- | --- | --- | --- | --- | --- | --- | --- | --- | --- | --- | --- |
|  |  |  |  | 95- | 95+ |  | Mean | Min. | 25% | median | 75% | 100% | P value |
| Land use |  |  |  |  |  |  |  |  |  |  |  |  |  |
| Non-irrigated | 00_04 | 147 | 57 | 24 | 65 | 0.0267 | 16 | 1 | 2 | 5 | 13 | 214 | <0.0001 |
| arable land | 10_35 | 23 | 83 | 25 | 98 |  | 42 | 1 | 12 | 17 | 52 | 203 |  |
|  | 62_73 | 14 | 79 | 26 | 100 |  | 37 | 3 | 8 | 12 | 40 | 141 |  |
| Sparsely vegetated | 0-4 | 169 | 59 | 27 | 67 | 0.0196 | 22 | 1 | 2 | 5 | 17 | 214 | 0.0952 |
| areas | 23-53 | 15 | 93 | 28 | 106 |  | 26 | 1 | 7 | 11 | 40 | 88 |  |
| Beaches, dunes, sand | 0 | 176 | 60 | 29 | 67 | 0.0249 | 21 | 1 | 2 | 5 | 17 | 214 | 0.0038 |
|  | 18 | 8 | 100 | 30 | 100 |  | 42 | 9 | 14 | 38 | 65 | 88 |  |
| Soil type |  |  |  |  |  |  |  |  |  |  |  |  |  |
| Coluvial | 0-6 | 130 | 65 | 32 | 73 | 0.4048 | 28 | 1 | 3 | 9 | 31 | 214 | 0.03453 |
|  | 24-51 | 23 | 61 | 33 | 81 |  | 10 | 1 | 3 | 5 | 14 | 39 |  |
|  | 81-100 | 31 | 52 | 34 | 69 |  | 6 | 1 | 1 | 3 | 5 | 26 |  |
| Ground type |  |  |  |  |  |  |  |  |  |  |  |  |  |
| Fluvisol | 00_01 | 123 | 55 | 36 | 64 | 0.0111 | 20 | 1 | 2 | 5 | 17 | 203 | 0.2015 |
|  | 14_28 | 15 | 60 | 37 | 85 |  | 23 | 1 | 1 | 8 | 10 | 141 |  |
|  | 72_100 | 46 | 80 | 38 | 92 |  | 27 | 1 | 5 | 9 | 26 | 214 |  |
| Petrocalcic xerosols | 00_00 | 107 | 60 | 39 | 69 | 0.5642 | 16 | 1 | 2 | 5 | 11 | 214 | 0.0036 |
|  | 02_09 | 16 | 56 | 40 | 81 |  | 14 | 1 | 1 | 10 | 17 | 57 |  |
|  | 72_100 | 61 | 67 | 41 | 79 |  | 34 | 1 | 5 | 13 | 36 | 203 |  |
